# HPV Vaccine utilization, Alberta 2008/09 – 2013/14 School year

**DOI:** 10.1186/s12879-016-1340-6

**Published:** 2016-01-13

**Authors:** Xianfang C. Liu, Christopher A. Bell, Kimberley A. Simmonds, Margaret L. Russell, Lawrence W. Svenson

**Affiliations:** 1 Department of Community Health Sciences, Cumming School of Medicine, University of Calgary, 3280 Hospital Drive NW, Calgary, AB T2N 4Z6 Canada; 2 Epidemiology and Surveillance Team, Alberta Ministry of Health, 23rd fl Telus Plaza NT, 10025 Jasper Avenue, Edmonton, AB T5J 1S6 Canada; 3 School of Public Health, University of Alberta, Edmonton, AB T6G 1C9 Canada

**Keywords:** Papilloma virus vaccines, HPV vaccination, *Vaccination/ut, Canada

## Abstract

**Background:**

In Canada both bivalent (bHPV) vaccine and quadrivalent HPV vaccine (qHPV) are authorized for use. In Alberta, while both vaccines are available for private purchase, only qHPV is publicly funded for school girls in grades 5 and 9 as of 2013. We describe HPV vaccine uptake in Alberta, by school year, from the start of the publicly funded program in the Fall of 2008 through to August 31^st^ 2014 and estimate the cumulative proportion of the female population who were vaccinated by the end of the 2013/14 school year.

**Methods:**

We used data from the Alberta Ministry of Health Immunization and Adverse Reaction to Immunization repository (publicly funded vaccine), the population-based Pharmaceutical Information Network information systems (privately purchased vaccine) for the period September 1, 2008 to August 31, 2014 and demographic data from the Alberta Health Care Insurance Plan Registry. We estimate vaccine uptake rates and explore them by attributes of person, time, place, vaccine funding, and number of doses received. We estimated the cumulative proportions of the female population (by age group and number of doses received) who had received HPV vaccine by the end of the 2013/14 school year.

**Results:**

Of the 169,259 unique individuals who received one or more doses of HPV vaccine over the period, 98.3 % were females, and 83.8 % received publicly funded vaccines. Vaccine uptake increased over the period. The cumulative proportion of females aged 9–26 years as of 2013/14 who had received two or more doses of vaccine was 34.3 %; for those aged 10–11 years 59.6 % and for those aged 14–15 years, 76.0 %. For those aged 9–26 years, 31.3 % had received three doses of vaccine.

**Conclusion:**

HPV vaccine uptake rates have increased in Alberta over the study period, most prominently among the age groups targeted by the publicly funded school-girl vaccine program.

## Background

Both quadrivalent (qHPV) and bivalent HPV vaccines (bHPV) have been authorized (licensed) for use in Canada. Only qHPV is publicly funded in the province of Alberta. The quadrivalent vaccine prevents infection from HPV types 6, 11, 16, and 18 (causal agents of anogenital warts, cervical and anogenital cancers, some head and neck cancers, anogenital cancers) and bHPV vaccine prevents infections from HPV types 16 and 18 (cervical and anogenital cancers, some head and neck cancers) [1].

In Canada, qHPV vaccine was authorized for use among females aged 9–26 years in 2006. In 2011, this authorization was expanded to include females aged 9–45 years and males aged 9–26 years. Additionally in 2011, bHPV vaccine was authorized for use among females aged 10–26 years. Canada’s National Advisory Committee on Immunization (NACI) has recommended the qHPV or bHPV vaccine for females aged 9–26 years and qHPV for males aged 9–26 years. Both vaccines were initially authorized to be administered in a three dose series; however NACI now recommends that they can be given as a two-dose series for immunocompetent persons aged 9–14 years [1].

In Alberta both bHPV and qHPV vaccines have been available for private purchase through pharmacies since Health Canada authorization was attained. However only qHPV vaccine has been publicly funded. Since the 2008/2009 school year (as part of Alberta’s Immunization Program) the vaccine has been part of the routine immunization schedule for grade 5 girls. In 2009/2010, the vaccine was made available to grade 9 girls for a three-year catch-up program [2]. In 2014/2015, the publicly funded qHPV vaccine program was expanded to included grade 5 boys with a four year catch-up program for grade 9 boys [3]. Alberta’s publicly funded HPV vaccination program administers vaccine as a 3-dose series [4], although the recent NACI recommendation for a 2-dose series is under consideration.

We describe the pattern of HPV vaccine utilization in Alberta from September 1, 2008 through to August 31, 2014, using population-based data from the Immunization and Adverse Reaction to Immunization Repository (Imm/ARI), and Alberta Pharmaceutical Information Network (PIN) information systems. We estimate vaccine uptake and explore it by attributes of person, time, place, vaccine funding, and number of doses received. We estimate the cumulative proportions of females aged 9–26 years, 10–11 years (most of those of this age are in grade 5), and 14–15 years (most of those of this age are in grade 9) that had received HPV vaccine as of the end of the 2013/14 school year.

## Methods

### Ethics and role of funding source

The study was approved by the University of Calgary Conjoint Health Research Ethics Board (REB15-0598/E-23776). Individual level consent was not required as all data were extracted and anonymized by data custodian prior to release to investigators. The funding source had no role in study design, collection, analysis or interpretation of data, report writing or publication decision.

### Data sources and data extraction

Alberta has a universal publicly funded health care system [5]. As described elsewhere [6], each individual is assigned a personal health number that acts as a unique lifetime identifier (ULI). The ULI is recorded each time a person accesses the healthcare system and this allows for deterministic linkage across multiple administrative data sets held by the Alberta Ministry of Health (Alberta Health). In the present study, we linked health insurance registration data (demographics) to Alberta’s immunization repository (Imm/ARI) and Pharmaceutical Information Network (PIN) information systems.

Imm/ARI contains a record for all publicly funded vaccines administered through public health clinics in the province. It contains information on the patient, vaccine administered, dose, date of immunization, location, and other information relevant to the immunization event. PIN contains a record for all prescriptions dispensed and includes information on the patient, date of dispense, location of pharmacy, and the pharmacologic product dispensed [i.e. drug identification number (DIN)]. In fall 2007, Alberta passed legislation requiring community pharmacies to submit data on all drugs dispensed [7]. It is estimated that over 95 % of pharmacologic products dispensed are included in PIN [8].

All data were extracted by employees of Alberta Health, the data custodian, and aggregated prior to release to the investigators. Immunization data for publicly and privately funded qHPV and bHPV vaccines as well as demographic data for immunized individuals from Imm/ARI and PIN were extracted, for the period September 1, 2008 to August 31, 2014. We estimated the number of immunized individuals by school year and population-level characteristics. For the purpose of this study, school year data captured immunization events between September 1 and August 31 of the following year (i.e., the 2012/13 school year captured immunization events between September 1, 2012 and August 31, 2013). The number of doses received for each individual was determined based on their last recorded dose over the interval (i.e., a person who received one dose of vaccine in 2008/09 and two doses in 2009/2010 is counted only once, and classified as having received dose 3 in 2009/2010). We extracted annual mid-year population estimates from the Alberta Health Care insurance plan registry (AHCIP) from 2009 to 2014. As described elsewhere [6], postal codes were used to classify persons as rural vs. urban residents and to assign income quintiles which were estimated from 2011 Canadian census data for Alberta.

### Data analysis

Data were analyzed using SAS 9.3 (SAS Institute Inc., Cary, NC 2011) and graphs (spline curve smoothing) created using Sigmaplot 13.0 (Systat Software, San Jose, CA 2013).

We explored the pattern of vaccine uptake rates for HPV vaccine by school year, age-group, gender, rural/urban residence, income quintile, type of vaccine funding (public or private), and total number of doses received (one, two, or three doses). Vaccine uptake rates were calculated as the percentage of the population who received one or more doses of HPV vaccine by school year and population-level characteristics. We estimated the cumulative proportion of females aged 9–26 years as of 2013/14 ever immunized with any HPV vaccine [i.e., bHPV or qHPV regardless of how the vaccine was funded (publicly funded, privately funded or mixed funding)]. We explored for associations between income quintile and type of vaccine funding (public vs. private funding); and for associations between rural/urban residence and time period in which immunized using a continuity adjusted chi square at alpha = 0.05.

## Results

### Demographic characteristics

Between September 1, 2008 and August 31, 2014, 169,259 individuals (98.3 % female) received one or more doses of any HPV vaccine (Table 1). Only 1 % of those who received HPV vaccine were dispensed bHPV. About 7 % (11,921) of those immunized received only one dose, 9.4 % (15,962) received only two doses, and 83.5 % (141,376) received three doses. The publicly funded school HPV vaccination program targets students in grade 5, most of whom would be aged 10 or 11 years: 48.2 % of those vaccinated. Most grade 9 students are aged 14 or 15 years, accounting for 30.5 % of persons vaccinated. The large majority of those immunized were aged 9–14 years (69.7 %), followed by those aged 15–20 years (20.6 %), and 21–26 years (6.2 %). About 3.6 % of those who received vaccine were aged 27–45 years and a very small number of persons were aged 46 years or older. Most people (83.8 %) received publicly funded vaccine; only 16.1 % received HPV vaccines through private purchase or mixed public-private (i.e. received at least one privately purchased dose and at least one publicly funded dose) funding (Table 1). There was an association between funding source for vaccine received (privately funded vaccine vs. publicly funded vaccine) and income. As can be seen from Table 1, among those who received privately funded vaccine, 50.4 % were in higher income quintiles (quintiles 4–5); compared to 42.6 % of those who received publicly funded vaccine (continuity adjusted chi square 1 df = 551.5861, *p* < 0.0001).

**Table 1:** Characteristics of persons who received one or more doses of HPV vaccine, 2008 - 2014

		Public funding	Private funding	Mixed funding	Total persons vaccinated N (%)^a^
Total [N, (%^a^)]		141,877 (83.8)	26,823 (15.8)	559 (0.3)	169,259
School year	2008/09	13,420	4,101	79	17,600 (10.4)
	2009/10	25,693	4,554	708	30,355 (17.9)
	2010/11	28,231	3,796	90	32,117 (19.0)
	2011/12	28,971	3,787	79	32,837 (19.4)
	2012/13	21,326	4,487	93	25,906 (15.3)
	2013/14	24,236	6,098	110	30,444 (18.0)
Age in years	9	397	10	11	408 (0.2)
	10^b^	50,787	43	16	50,846 (30.0)
	11^b^	30,670	67	10	30,747 (18.2)
	12	2,734	121	15	2,870 (1.7)
	13	1,730	200	18	1,948 (1.2)
	14^c^	30,615	364	108	31,087 (18.4)
	15^c^	19,504	954	91	20,549 (12.1)
	16	1,686	1,608	52	3,346 (2.0)
	17	1,200	2,151	54	3,405 (2.0)
	18	740	2,259	43	3,042 (1.8)
	19	294	1,953	31	2,278 (1.3)
	20	229	1,905	25	2,159 (1.3)
	21 to 26	1,150	9,301	76	10,527 (6.2)
	27 to 45	135	5,474	19	5,628 (3.3)
	>45	4	413	0	417 (0.3)
Gender	Female	141,661	24,180	545	166,386 (98.3)
	Male	216	2,643	14	2,873 (1.7)
Doses received	One dose	4,206	7,715	0	11,921 (7.0)
	Two doses	9,599	6,316	47	15,962 (9.4)
	Three doses	128,072	12,792	512	141,376 (83.5)
Rural/Urban Residence	Rural	29,446	3,804	163	33,413 (19.8)
	Urban	112,431	23,019	396	135,846 (80.3)
Income quintile	Q1	27,716	3,556	100	31,372 (18.5)
	Q2	28,184	4,664	106	32,954 (19.5)
	Q3	25,515	5,089	91	30,695 (18.1)
	Q4	30,991	6,591	140	37,722 (22.3)
	Q5	29,471	6,923	122	36,516 (21.6)
Type of HPV vaccine	qHPV	141,851	25,141	536	167,528 (99.0)
	bHPV	11	1,682	23	1,716 (1.0)
	U/K^d^	15	0	0	15 (0)

^a^Some percentages may not add to 100 due to rounding

^b^Most children aged 10 or 11 years are grade 5 students

^c^Most children aged 14 or 15 years are grade 9 students

^d^U/K = unknown

### Vaccine uptake rates

**Fig. 1:**
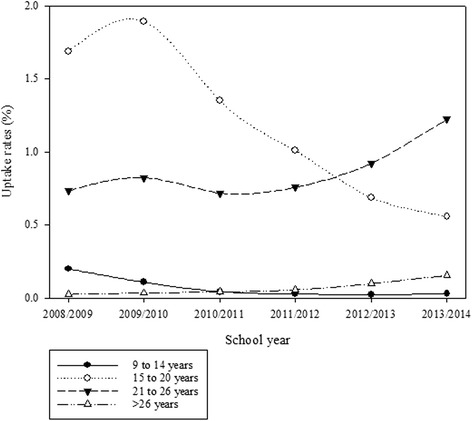
Age-group-specific uptake rates by school year for privately funded HPV vaccines among females aged 9 years or older

**Fig. 2:**
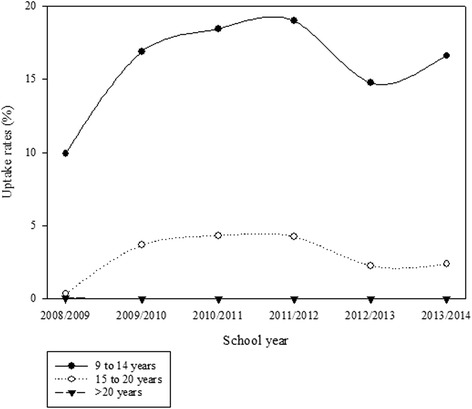
Age-group-specific uptake rates by school year for publicly funded HPV vaccines among females aged 9 years or older

The patterns of vaccine uptake rates were examined (Figs. 1 and 2). Uptake rates for privately funded HPV vaccines were highest among females aged 15–20 years between 2008/09 (1.7 %) and 2011/12 (1.0 %) (Fig. 1). The shape of the curves show that uptake rates in this age-group have been decreasing since 2009/10 while uptake rates among those aged >20 years have increased. By 2013/14, uptake rates for privately funded vaccine were highest among females aged 21–26 years (1.2 %). Among females who received publicly funded vaccines, uptake rates were highest among females aged 9–14 years (9.9 % in 2008/09; 19.0 % in 2011/12; and 16.6 % in 2013/14) (Fig. 2). Uptake rates were lower among those aged 15–20 years (0.4 % in 2008/09; 4.3 % in 2011/12; and 2.4 % in 2013/14). Uptake rates were less than 0.5 % among females aged >20 years.

Among males aged 9 years or older, vaccine uptake for privately funded vaccines across all age-groups increased over the period. Within each school year, uptake rates were highest among those aged 15–20 years. Males aged 21–26 years had the second highest uptake rates while those aged 27 years or older had the lowest uptake (data not shown).

Among females aged 9–14 years who received publicly funded HPV vaccine, uptake rates for three doses were higher than for only one or only two doses over the period (data not shown). Uptake rates for three doses greatly increased from 2008/09 to 2009/10, and remained consistently high until 2012/13. Uptake rates for only one or only two doses increased over the period. In 2013/14, the one dose uptake rate was 0.7 %, two doses 1.9 %, and three doses 14.0 %.

**Fig. 3:**
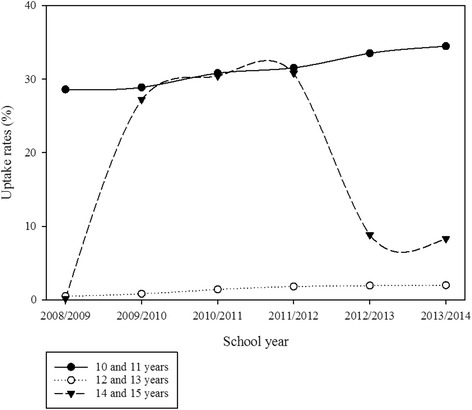
Age-specific uptake rates for three doses of HPV vaccine among females aged 9–15 years who received publicly funded HPV vaccine by school yea

Figure 3 shows age-specific uptake rates for three doses of publicly funded HPV vaccine among females aged 10–15 years by school year. Most females who received three doses were aged 10 and 11 years (grade 5) or 14 and 15 years (grade 9). Uptake rates increased among females aged 10 and 11 years over the period. Among females aged 14 or 15 years, uptake rates increased between 2009/10 and 2011/12 but decreased in 2012/13. The pattern of age-specific uptake rates for the receipt of only one or two doses was similar (not shown).

There was an association between time periods within the interval studied and vaccine uptake by rural/urban residents (Yates corrected Chi-square = 319.667, df = 1, two-tailed *p*-value <0.0001). Vaccine uptake was similar for urban and rural residents for the earlier part of the interval (2008/09- 2011/12). However in the period 2012/13-2013/14, uptake was slightly higher among urban compared to rural residents (data not shown).

### Cumulative proportion of females immunized by the end of 2013/14

**Figure 4:**
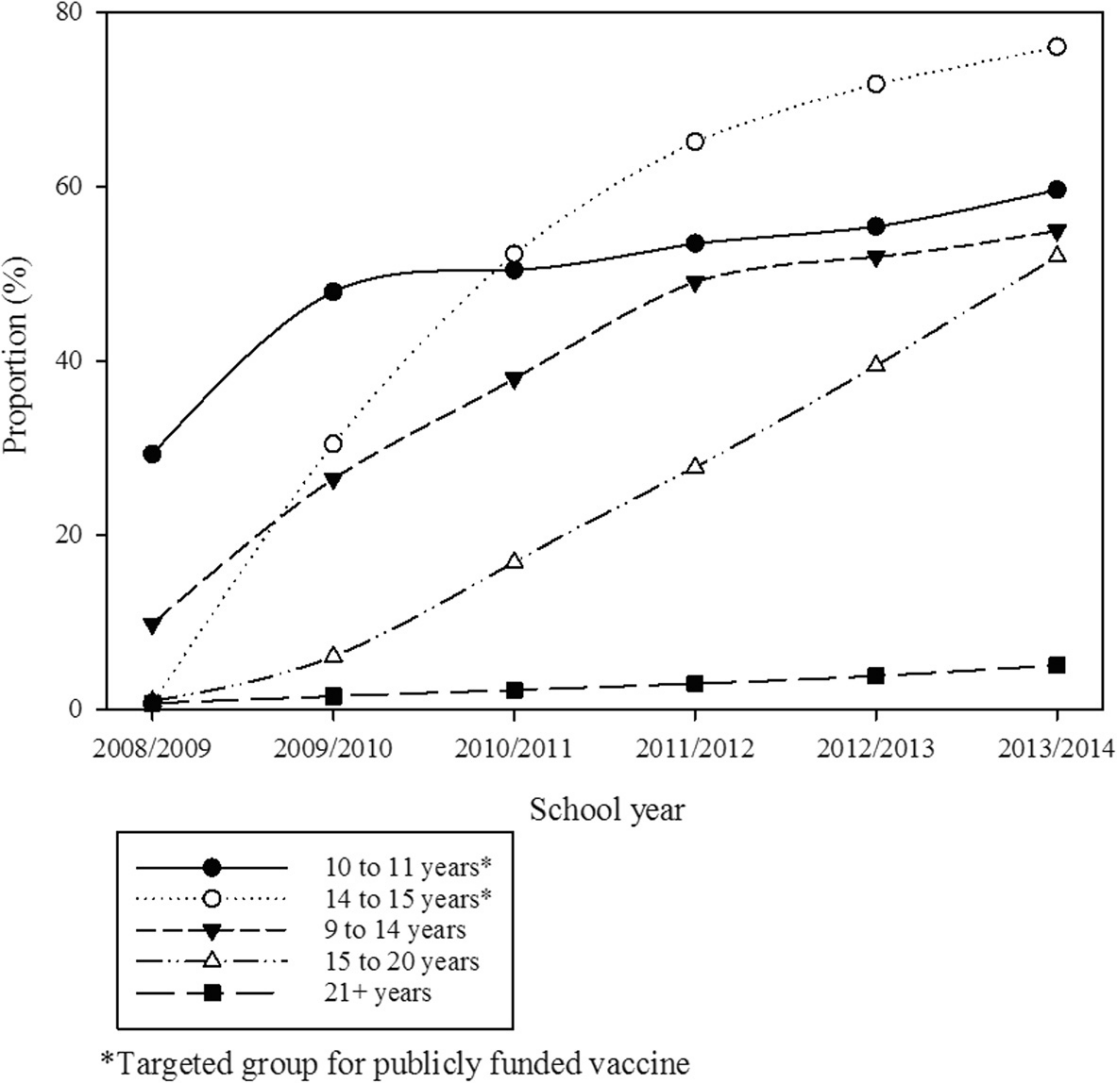
Cumulative proportion of females immunized (2 or more doses of any HPV vaccine, regardless of funding mechanism for vaccine) by school year and age groups

The cumulative proportion of females aged 9–26 years as of 2013/14 who had received only one HPV vaccine dose was 2.0 %, one or more doses of HPV vaccine 36.3 %, 34.3 % for those who had obtained 2 or more doses and 31.3 % for 3 doses. For those aged 10–11 years as of 2013/14, the respective cumulative proportions were 1.8 % (only 1 dose), 61.4 % (one or more doses); 59.6 % (2 or more doses) and 54.8 % for 3 doses. Similarly, for those girls aged 14–15 years as of 2013/14, the cumulative proportion who received only one dose 1.9 %, one or more doses 78.0 %; 76.0 % (2 or more doses) and 71.8 % (3 doses). For those aged 9–14, 15–20, and 21–26 years, the cumulative proportion of females who received two or more doses were 55.0 %, 52.0 %, and 5.1 %, respectively as of 2013/14 (Fig. 4).

## Discussion

This study had the strengths of population based data, including capture of both publicly funded and privately purchased HPV vaccine data. HPV vaccine uptake rates increased in Alberta between 2008/09 and 2011/12 but have fluctuated in recent years. The majority of people received publicly funded vaccines, and the impact of public funding is particularly seen among the age groups targeted by the publicly funded school girl immunization program.

When grade 9 girls became eligible for publicly funded vaccines in the 2009/10 school year, there was an increase in uptake rates among those aged 14–15 years, followed by a decline in 2012/13. We posit that completion of the three-year catch-up for grade 9 girls in the publicly funded HPV immunization program was responsible for this decrease, rather than other reasons such as public concern about vaccine adverse effects. The date for the decline corresponded with the end of the catch-up program. We did not see a similar large decrease in uptake rates among girls aged 10 and 11 years or aged 14 and 15 years who received publicly funded vaccines, nor among females who received privately funded vaccines which is what might be anticipated if there had been a change in perceptions of adverse effects from vaccination.

Increased publicly funded HPV vaccine uptake rates have coincided with decreased privately funded HPV vaccine uptake rates among females aged 15–20 years between 2008/09 and 2011/12. The majority of females who received privately funded vaccines were aged 21–26 years and thus ineligible for publicly funded vaccines. This trend was not surprising since the cost of HPV vaccine is around $150/dose [9].

Increased vaccine uptake rates for privately funded vaccines among females aged 21–26 years and among males suggest that demand for HPV vaccine is increasing. This is likely due to individuals becoming aware of the vaccine due to counselling from health care providers, media or direct to consumer advertising, wider acceptance, or availability. We expect to see a large increase in vaccine uptake among males aged 10–11 and 14–15 years starting in 2014, due to the expansion of the publicly funded program to grade 5 males (with catch up for grade 9 males) starting in 2014.

Publicly funded HPV vaccines are delivered by public health nurses. The program is designed to permit administration of three doses during the school year. Classes run from September to June of the next year, where the first HPV vaccine dose is typically dispensed around October. The earliest date that the second dose could be delivered is two months later in November or December, and the third dose could be delivered in March or April of the following year. For this reason, if the number of immunizations is not tied to the individual and reporting based upon completion of a series, vaccine uptake estimates that consider ‘school year’ rather than ‘calendar year’ time periods may be misleading if used as indicators of program acceptability.

Based on current recommendations for a three dose HPV vaccine series, receipt of three doses is considered “vaccine completion” [1]; thus the cumulative proportion of females aged 9–26 years as of 2013/14 who had completed the series was 31.3 %, for those aged 10–11 years, 54.8 % and 71.8 % for those aged 14–15 years If Alberta switched to a two-dose HPV vaccine, we should expect to observe an increase in the cumulative proportion of the population who complete the vaccine series because fewer doses would be required. As of 2013/14, the cumulative proportion of females aged 9–26 years who had received 2 more or more doses was 34.3 %.

Brisson modeled the incremental effectiveness of immunizing males and found that the benefits of immunizing males may be limited when vaccine coverage among young girls is moderate to high due to the indirect protective effect from immunizing females [10]. Brisson and colleagues [10] have identified that the incremental effect of adding boys to an HPV vaccination program may increase under scenarios where a proportion of girls do not complete the vaccination series. As the cumulative proportion of females aged 9–26 years as of 2013/14 who had received two or more doses of vaccine was only 34 %, we anticipate seeing greater incremental benefits and disease reductions by immunizing both males and females in Alberta, rather than females alone.

There were potential limitations to this study. PIN vaccine dispensing data assumes that people who were dispensed HPV vaccine actually received the vaccine. We think that those dispensed this vaccine are likely to follow-up with a care provider for vaccine administration: the high price to consumer [9] might be a strong incentive for such follow-through. We may have misclassified some persons as having received HPV vaccine when another vaccine was received or data was incorrectly entered when vaccine dispensing information was reported. We think this unlikely, as there are a number of business rules in place in the Imm/ARI system, but cannot rule out the possibility. We estimated the proportion of Albertans that received HPV vaccines by the end of 2013/14 while adjusting for deaths and outmigration. However, Alberta residents who were immunized out of province were not accounted for. This will underestimate the proportion of those who were vaccinated within each age-group.

## Conclusion

HPV vaccine uptake rates have increased among the targeted population in Alberta over the period. Females aged 9–14 years who were targeted had the highest uptake rates for any dose of publicly funded vaccine. Females aged 10–11 years also had the highest uptake rates for three doses of publicly funded vaccine. This is consistent with the targeting of the publicly funded immunization program.

## Abbreviations

HPV: Human papilloma virus
qHPV: quadrivalent human papilloma virus vaccine
bHPV: Bivalent human papilloma virus vaccine
Imm/ARI: Immunization and Adverse Reaction to Immunization Repository
PIN: Alberta Pharmaceutical Information Network
ULI: Unique lifetime identifier
Alberta Health: Ministry of Health
DIN: Drug identification number
AHCIP: Alberta Health Care insurance plan registry
Df: Degree of freedom
